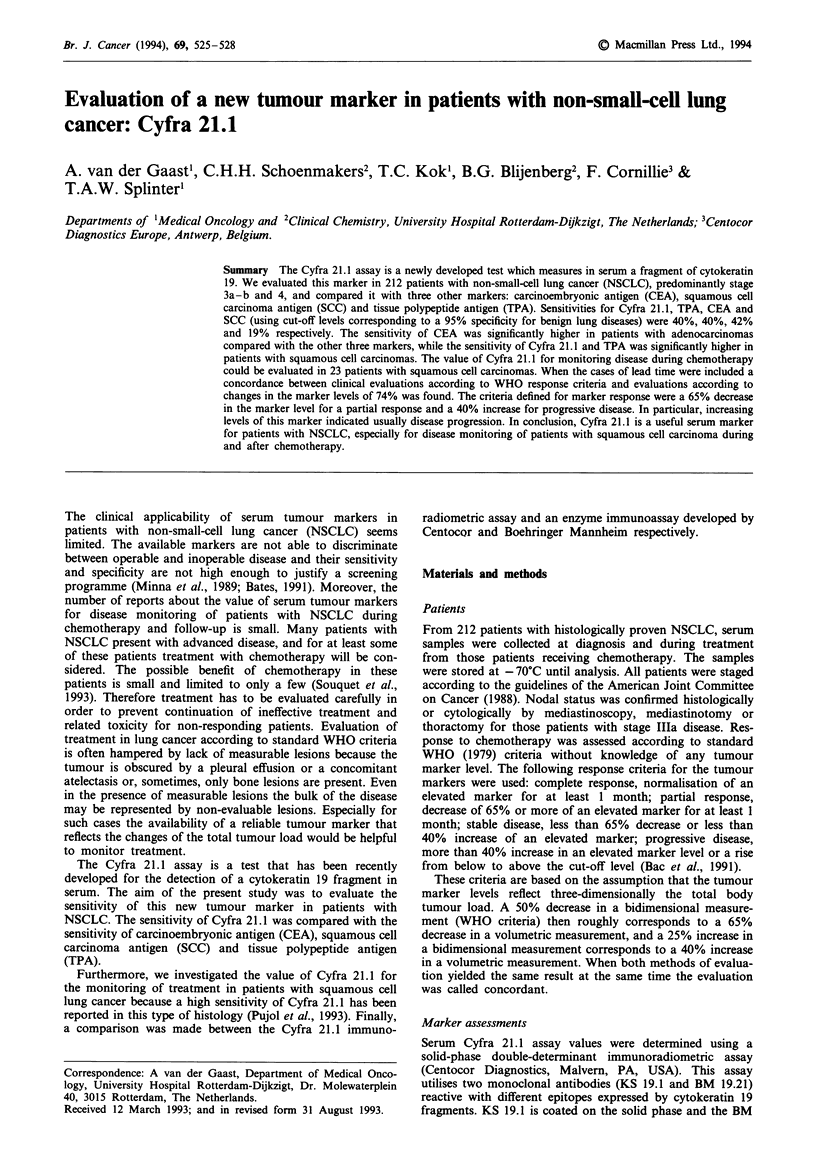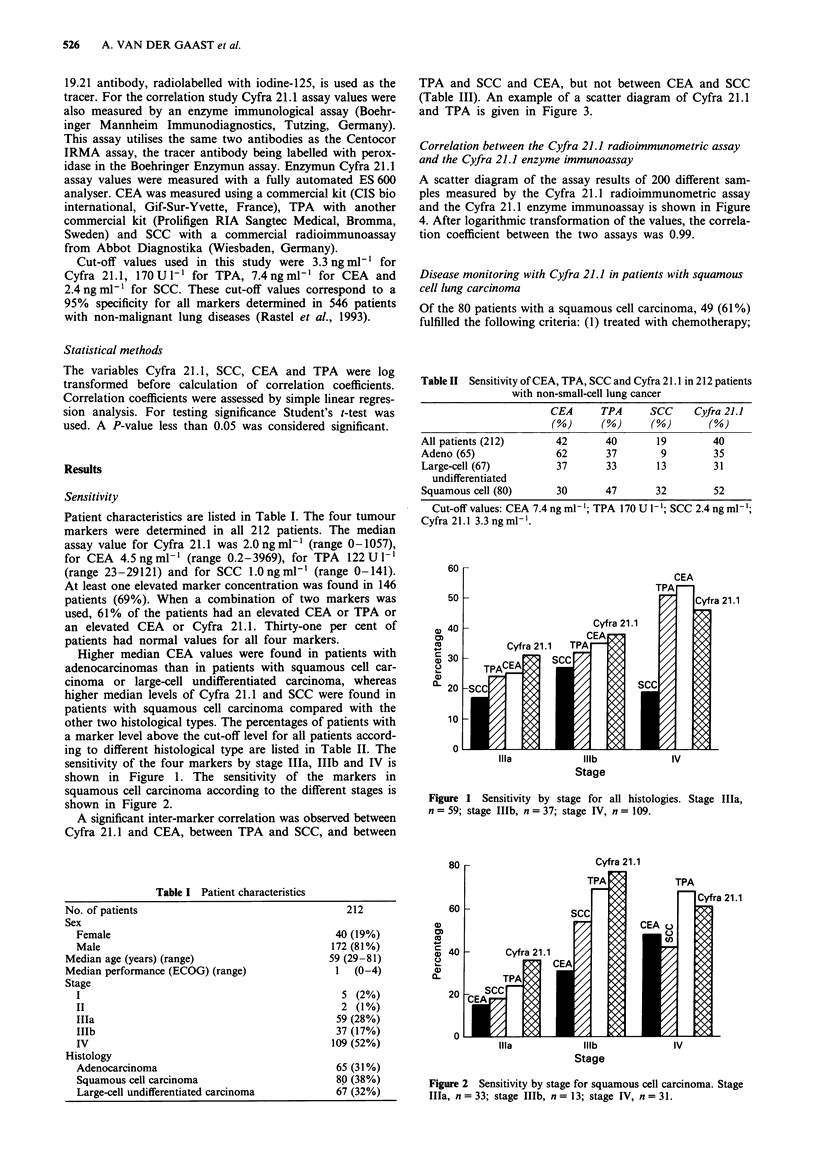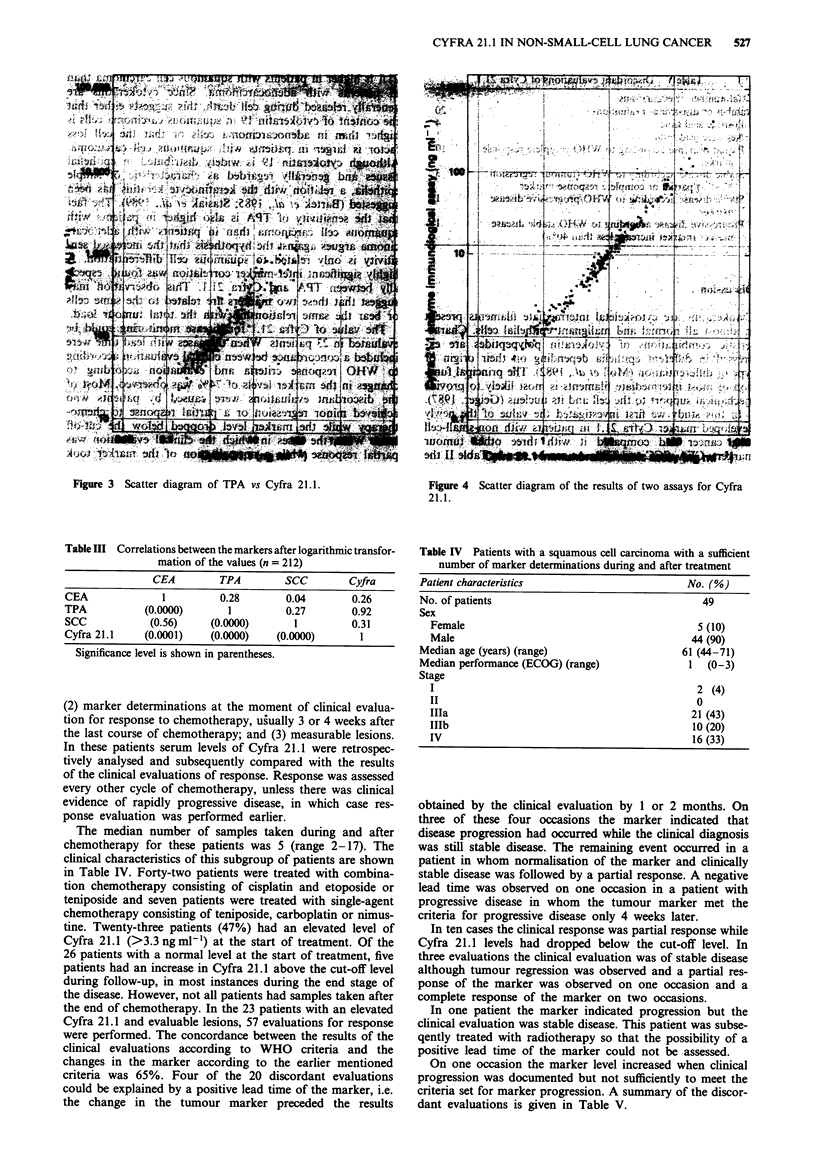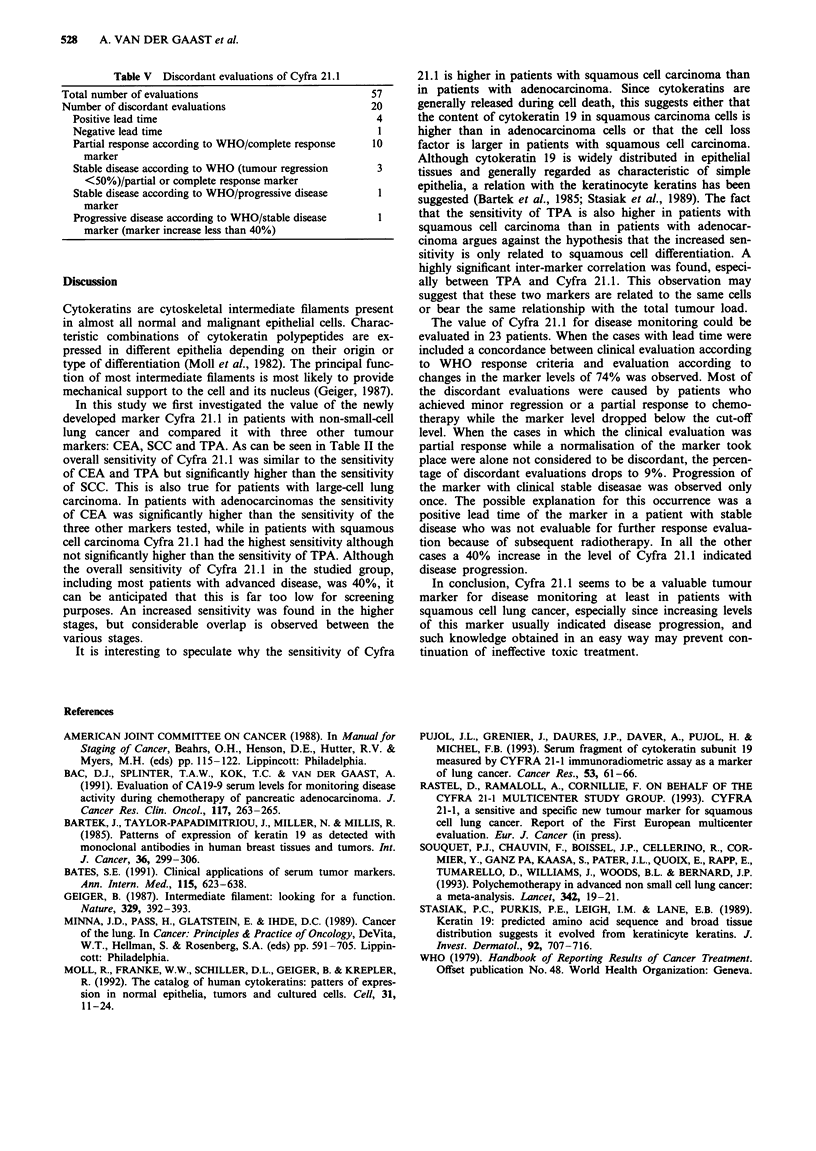# Evaluation of a new tumour marker in patients with non-small-cell lung cancer: Cyfra 21.1.

**DOI:** 10.1038/bjc.1994.95

**Published:** 1994-03

**Authors:** A. van der Gaast, C. H. Schoenmakers, T. C. Kok, B. G. Blijenberg, F. Cornillie, T. A. Splinter

**Affiliations:** Department of Medical Oncology, University Hospital Rotterdam-Dijkzigt, The Netherlands.

## Abstract

The Cyfra 21.1 assay is a newly developed test which measures in serum a fragment of cytokeratin 19. We evaluated this marker in 212 patients with non-small-cell lung cancer (NSCLC), predominantly stage 3a-b and 4, and compared it with three other markers: carcinoembryonic antigen (CEA), squamous cell carcinoma antigen (SCC) and tissue polypeptide antigen (TPA). Sensitivities for Cyfra 21.1, TPA, CEA and SCC (using cut-off levels corresponding to a 95% specificity for benign lung diseases) were 40%, 40%, 42% and 19% respectively. The sensitivity of CEA was significantly higher in patients with adenocarcinomas compared with the other three markers, while the sensitivity of Cyfra 21.1 and TPA was significantly higher in patients with squamous cell carcinomas. The value of Cyfra 21.1 for monitoring disease during chemotherapy could be evaluated in 23 patients with squamous cell carcinomas. When the cases of lead time were included a concordance between clinical evaluations according to WHO response criteria and evaluations according to changes in the marker levels of 74% was found. The criteria defined for marker response were a 65% decrease in the marker level for a partial response and a 40% increase for progressive disease. In particular, increasing levels of this marker indicated usually disease progression. In conclusion, Cyfra 21.1 is a useful serum marker for patients with NSCLC, especially for disease monitoring of patients with squamous cell carcinoma during and after chemotherapy.


Br. J. Cancer (1994), 69, 525-528 © Macmillan Press Ltd., 1994

### Evaluation of a new tumour marker in patients with non-small-cell lung cancer: Cyfra 21.1

A. van der Gaast[1], C.H.H. Schoenmakers[2], T.C. Kok[1], B.G. Blijenberg[2], F. Cornillie[3] & T.A.W. Splinter[1]

*Departments of [1]Medical Oncology and [2]Clinical Chemistry, University Hospital Rotterdam-Dijkzigt, The Netherlands; [3]Centocor Diagnostics Europe, Antwerp, Belgium.*

**Summary** The Cyfra 21.1 assay is a newly developed test which measures in serum a fragment of cytokeratin 19. We evaluated this marker in 212 patients with non-small-cell lung cancer (NSCLC), predominantly stage 3a-b and 4, and compared it with three other markers: carcinoembryonic antigen (CEA), squamous cell carcinoma antigen (SCC) and tissue polypeptide antigen (TPA). Sensitivities for Cyfra 21.1, TPA, CEA and SCC (using cut-off levels corresponding to a 95% specificity for benign lung diseases) were 40%, 40%, 42% and 19% respectively. The sensitivity of CEA was significantly higher in patients with adenocarcinomas compared with the other three markers, while the sensitivity of Cyfra 21.1 and TPA was significantly higher in patients with squamous cell carcinomas. The value of Cyfra 21.1 for monitoring disease during chemotherapy could be evaluated in 23 patients with squamous cell carcinomas. When the cases of lead time were included a concordance between clinical evaluations according to WHO response criteria and evaluations according to changes in the marker levels of 74% was found. The criteria defined for marker response were a 65% decrease in the marker level for a partial response and a 40% increase for progressive disease. In particular, increasing levels of this marker indicated usually disease progression. In conclusion, Cyfra 21.1 is a useful serum marker for patients with NSCLC, especially for disease monitoring of patients with squamous cell carcinoma during and after chemotherapy.

The clinical applicability of serum tumour markers in patients with non-small-cell lung cancer (NSCLC) seems limited. The available markers are not able to discriminate between operable and inoperable disease and their sensitivity and specificity are not high enough to justify a screening programme (Minna *et al.*, 1989; Bates, 1991). Moreover, the number of reports about the value of serum tumour markers for disease monitoring of patients with NSCLC during chemotherapy and follow-up is small. Many patients with NSCLC present with advanced disease, and for at least some of these patients treatment with chemotherapy will be considered. The possible benefit of chemotherapy in these patients is small and limited to only a few (Souquet *et al.*, 1993). Therefore treatment has to be evaluated carefully in order to prevent continuation of ineffective treatment and related toxicity for non-responding patients. Evaluation of treatment in lung cancer according to standard WHO criteria is often hampered by lack of measurable lesions because the tumour is obscured by a pleural effusion or a concomitant atelectasis or, sometimes, only bone lesions are present. Even in the presence of measurable lesions the bulk of the disease may be represented by non-evaluable lesions. Especially for such cases the availability of a reliable tumour marker that reflects the changes of the total tumour load would be helpful to monitor treatment.

The Cyfra 21.1 assay is a test that has been recently developed for the detection of a cytokeratin 19 fragment in serum. The aim of the present study was to evaluate the sensitivity of this new tumour marker in patients with NSCLC. The sensitivity of Cyfra 21.1 was compared with the sensitivity of carcinoembryonic antigen (CEA), squamous cell carcinoma antigen (SCC) and tissue polypeptide antigen (TPA).

Furthermore, we investigated the value of Cyfra 21.1 for the monitoring of treatment in patients with squamous cell lung cancer because a high sensitivity of Cyfra 21.1 has been reported in this type of histology (Pujol *et al.*, 1993). Finally, a comparison was made between the Cyfra 21.1 immunoradiometric assay and an enzyme immunoassay developed by Centocor and Boehringer Mannheim respectively.

Correspondence: A van der Gaast, Department of Medical Oncology, University Hospital Rotterdam-Dijkzigt, Dr. Molewaterplein 40, 3015 Rotterdam, The Netherlands.
Received 12 March 1993; and in revised form 31 August 1993.

### Materials and methods

### *Patients*

From 212 patients with histologically proven NSCLC, serum samples were collected at diagnosis and during treatment from those patients receiving chemotherapy. The samples were stored at −70°C until analysis. All patients were staged according to the guidelines of the American Joint Committee on Cancer (1988). Nodal status was confirmed histologically or cytologically by mediastinoscopy, mediastinotomy or thoractomy for those patients with stage IIIa disease. Response to chemotherapy was assessed according to standard WHO (1979) criteria without knowledge of any tumour marker level. The following response criteria for the tumour markers were used: complete response, normalisation of an elevated marker for at least 1 month; partial response, decrease of 65% or more of an elevated marker for at least 1 month; stable disease, less than 65% decrease or less than 40% increase of an elevated marker; progressive disease, more than 40% increase in an elevated marker level or a rise from below to above the cut-off level (Bac *et al.*, 1991).

These criteria are based on the assumption that the tumour marker levels reflect three-dimensionally the total body tumour load. A 50% decrease in a bidimensional measurement (WHO criteria) then roughly corresponds to a 65% decrease in a volumetric measurement, and a 25% increase in a bidimensional measurement corresponds to a 40% increase in a volumetric measurement. When both methods of evaluation yielded the same result at the same time the evaluation was called concordant.

### *Marker assessments*

Serum Cyfra 21.1 assay values were determined using a solid-phase double-determinant immunoradiometric assay (Centocor Diagnostics, Malvern, PA, USA). This assay utilises two monoclonal antibodies (KS 19.1 and BM 19.21) reactive with different epitopes expressed by cytokeratin 19 fragments. KS 19.1 is coated on the solid phase and the BM

19.21 antibody, radiolabelled with iodine-125, is used as the tracer. For the correlation study Cyfra 21.1 assay values were also measured by an enzyme immunological assay (Boehringer Mannheim Immunodiagnostics, Tutzing, Germany). This assay utilises the same two antibodies as the Centocor IRMA assay, the tracer antibody being labelled with peroxidase in the Boehringer Enzymun assay. Enzymun Cyfra 21.1 assay values were measured with a fully automated ES 600 analyser. CEA was measured using a commercial kit (CIS bio international, Gif-Sur-Yvette, France), TPA with another commercial kit (Prolifigen RIA Sangtec Medical, Bromma, Sweden) and SCC with a commercial radioimmunoassay from Abbot Diagnostika (Wiesbaden, Germany).

Cut-off values used in this study were 3.3 ng $ml^{-1}$ for Cyfra 21.1, 170 U $l^{-1}$ for TPA, 7.4 ng $ml^{-1}$ for CEA and 2.4 ng $ml^{-1}$ for SCC. These cut-off values correspond to a 95% specificity for all markers determined in 546 patients with non-malignant lung diseases (Rastel et al., 1993).

*Statistical methods*

The variables Cyfra 21.1, SCC, CEA and TPA were log transformed before calculation of correlation coefficients. Correlation coefficients were assessed by simple linear regression analysis. For testing significance Student's *t*-test was used. A *P*-value less than 0.05 was considered significant.

### Results

*Sensitivity*

Patient characteristics are listed in Table I. The four tumour markers were determined in all 212 patients. The median assay value for Cyfra 21.1 was 2.0 ng $ml^{-1}$ (range 0–1057), for CEA 4.5 ng $ml^{-1}$ (range 0.2–3969), for TPA 122 U $l^{-1}$ (range 23–29121) and for SCC 1.0 ng $ml^{-1}$ (range 0–141). At least one elevated marker concentration was found in 146 patients (69%). When a combination of two markers was used, 61% of the patients had an elevated CEA or TPA or an elevated CEA or Cyfra 21.1. Thirty-one per cent of patients had normal values for all four markers.

Higher median CEA values were found in patients with adenocarcinomas than in patients with squamous cell carcinoma or large-cell undifferentiated carcinoma, whereas higher median levels of Cyfra 21.1 and SCC were found in patients with squamous cell carcinoma compared with the other two histological types. The percentages of patients with a marker level above the cut-off level for all patients according to different histological type are listed in Table II. The sensitivity of the four markers by stage IIIa, IIIb and IV is shown in Figure 1. The sensitivity of the markers in squamous cell carcinoma according to the different stages is shown in Figure 2.

A significant inter-marker correlation was observed between Cyfra 21.1 and CEA, between TPA and SCC, and between TPA and SCC and CEA, but not between CEA and SCC (Table III). An example of a scatter diagram of Cyfra 21.1 and TPA is given in Figure 3.

*Correlation between the Cyfra 21.1 radioimmunometric assay and the Cyfra 21.1 enzyme immunoassay*

A scatter diagram of the assay results of 200 different samples measured by the Cyfra 21.1 radioimmunometric assay and the Cyfra 21.1 enzyme immunoassay is shown in Figure 4. After logarithmic transformation of the values, the correlation coefficient between the two assays was 0.99.

*Disease monitoring with Cyfra 21.1 in patients with squamous cell lung carcinoma*

Of the 80 patients with a squamous cell carcinoma, 49 (61%) fulfilled the following criteria: (1) treated with chemotherapy;

**Table I** Patient characteristics

| | |
|---|---|
| No. of patients | 212 |
| Sex | |
| Female | 40 (19%) |
| Male | 172 (81%) |
| Median age (years) (range) | 59 (29–81) |
| Median performance (ECOG) (range) | 1 (0–4) |
| Stage | |
| I | 5 (2%) |
| II | 2 (1%) |
| IIIa | 59 (28%) |
| IIIb | 37 (17%) |
| IV | 109 (52%) |
| Histology | |
| Adenocarcinoma | 65 (31%) |
| Squamous cell carcinoma | 80 (38%) |
| Large-cell undifferentiated carcinoma | 67 (32%) |

**Table II** Sensitivity of CEA, TPA, SCC and Cyfra 21.1 in 212 patients with non-small-cell lung cancer

| | *CEA (%)* | *TPA (%)* | *SCC (%)* | *Cyfra 21.1 (%)* |
|---|---|---|---|---|
| All patients (212) | 42 | 40 | 19 | 40 |
| Adeno (65) | 62 | 37 | 9 | 35 |
| Large-cell (67) undifferentiated | 37 | 33 | 13 | 31 |
| Squamous cell (80) | 30 | 47 | 32 | 52 |

Cut-off values: CEA 7.4 ng $ml^{-1}$; TPA 170 U $l^{-1}$; SCC 2.4 ng $ml^{-1}$; Cyfra 21.1 3.3 ng $ml^{-1}$.

**Figure 1** Sensitivity by stage for all histologies. Stage IIIa, $n = 59$; stage IIIb, $n = 37$; stage IV, $n = 109$.

**Figure 2** Sensitivity by stage for squamous cell carcinoma. Stage IIIa, $n = 33$; stage IIIb, $n = 13$; stage IV, $n = 31$.

**Figure 3** Scatter diagram of TPA *vs* Cyfra 21.1.

**Figure 4** Scatter diagram of the results of two assays for Cyfra 21.1.

**Table III** Correlations between the markers after logarithmic transformation of the values ($n = 212$)

| | *CEA* | *TPA* | *SCC* | *Cyfra* |
|---|---|---|---|---|
| CEA | 1 | 0.28 | 0.04 | 0.26 |
| TPA | (0.0000) | 1 | 0.27 | 0.92 |
| SCC | (0.56) | (0.0000) | 1 | 0.31 |
| Cyfra 21.1 | (0.0001) | (0.0000) | (0.0000) | 1 |

Significance level is shown in parentheses.

(2) marker determinations at the moment of clinical evaluation for response to chemotherapy, usually 3 or 4 weeks after the last course of chemotherapy; and (3) measurable lesions. In these patients serum levels of Cyfra 21.1 were retrospectively analysed and subsequently compared with the results of the clinical evaluations of response. Response was assessed every other cycle of chemotherapy, unless there was clinical evidence of rapidly progressive disease, in which case response evaluation was performed earlier.

The median number of samples taken during and after chemotherapy for these patients was 5 (range 2–17). The clinical characteristics of this subgroup of patients are shown in Table IV. Forty-two patients were treated with combination chemotherapy consisting of cisplatin and etoposide or teniposide and seven patients were treated with single-agent chemotherapy consisting of teniposide, carboplatin or nimustine. Twenty-three patients (47%) had an elevated level of Cyfra 21.1 (>3.3 ng ml$^{-1}$) at the start of treatment. Of the 26 patients with a normal level at the start of treatment, five patients had an increase in Cyfra 21.1 above the cut-off level during follow-up, in most instances during the end stage of the disease. However, not all patients had samples taken after the end of chemotherapy. In the 23 patients with an elevated Cyfra 21.1 and evaluable lesions, 57 evaluations for response were performed. The concordance between the results of the clinical evaluations according to WHO criteria and the changes in the marker according to the earlier mentioned criteria was 65%. Four of the 20 discordant evaluations could be explained by a positive lead time of the marker, i.e. the change in the tumour marker preceded the results

**Table IV** Patients with a squamous cell carcinoma with a sufficient number of marker determinations during and after treatment

| *Patient characteristics* | *No. (%)* |
|---|---|
| No. of patients | 49 |
| Sex | |
| Female | 5 (10) |
| Male | 44 (90) |
| Median age (years) (range) | 61 (44–71) |
| Median performance (ECOG) (range) | 1 (0–3) |
| Stage | |
| I | 2 (4) |
| II | 0 |
| IIIa | 21 (43) |
| IIIb | 10 (20) |
| IV | 16 (33) |

obtained by the clinical evaluation by 1 or 2 months. On three of these four occasions the marker indicated that disease progression had occurred while the clinical diagnosis was still stable disease. The remaining event occurred in a patient in whom normalisation of the marker and clinically stable disease was followed by a partial response. A negative lead time was observed on one occasion in a patient with progressive disease in whom the tumour marker met the criteria for progressive disease only 4 weeks later.

In ten cases the clinical response was partial response while Cyfra 21.1 levels had dropped below the cut-off level. In three evaluations the clinical evaluation was of stable disease although tumour regression was observed and a partial response of the marker was observed on one occasion and a complete response of the marker on two occasions.

In one patient the marker indicated progression but the clinical evaluation was stable disease. This patient was subseqently treated with radiotherapy so that the possibility of a positive lead time of the marker could not be assessed.

On one occasion the marker level increased when clinical progression was documented but not sufficiently to meet the criteria set for marker progression. A summary of the discordant evaluations is given in Table V.

**Table V** Discordant evaluations of Cyfra 21.1

| | |
|---|---|
| Total number of evaluations | 57 |
| Number of discordant evaluations | 20 |
| Positive lead time | 4 |
| Negative lead time | 1 |
| Partial response according to WHO/complete response marker | 10 |
| Stable disease according to WHO (tumour regression <50%)/partial or complete response marker | 3 |
| Stable disease according to WHO/progressive disease marker | 1 |
| Progressive disease according to WHO/stable disease marker (marker increase less than 40%) | 1 |

### Discussion

Cytokeratins are cytoskeletal intermediate filaments present in almost all normal and malignant epithelial cells. Characteristic combinations of cytokeratin polypeptides are expressed in different epithelia depending on their origin or type of differentiation (Moll et al., 1982). The principal function of most intermediate filaments is most likely to provide mechanical support to the cell and its nucleus (Geiger, 1987).

In this study we first investigated the value of the newly developed marker Cyfra 21.1 in patients with non-small-cell lung cancer and compared it with three other tumour markers: CEA, SCC and TPA. As can be seen in Table II the overall sensitivity of Cyfra 21.1 was similar to the sensitivity of CEA and TPA but significantly higher than the sensitivity of SCC. This is also true for patients with large-cell lung carcinoma. In patients with adenocarcinomas the sensitivity of CEA was significantly higher than the sensitivity of the three other markers tested, while in patients with squamous cell carcinoma Cyfra 21.1 had the highest sensitivity although not significantly higher than the sensitivity of TPA. Although the overall sensitivity of Cyfra 21.1 in the studied group, including most patients with advanced disease, was 40%, it can be anticipated that this is far too low for screening purposes. An increased sensitivity was found in the higher stages, but considerable overlap is observed between the various stages.

It is interesting to speculate why the sensitivity of Cyfra 21.1 is higher in patients with squamous cell carcinoma than in patients with adenocarcinoma. Since cytokeratins are generally released during cell death, this suggests either that the content of cytokeratin 19 in squamous carcinoma cells is higher than in adenocarcinoma cells or that the cell loss factor is larger in patients with squamous cell carcinoma. Although cytokeratin 19 is widely distributed in epithelial tissues and generally regarded as characteristic of simple epithelia, a relation with the keratinocyte keratins has been suggested (Bartek et al., 1985; Stasiak et al., 1989). The fact that the sensitivity of TPA is also higher in patients with squamous cell carcinoma than in patients with adenocarcinoma argues against the hypothesis that the increased sensitivity is only related to squamous cell differentiation. A highly significant inter-marker correlation was found, especially between TPA and Cyfra 21.1. This observation may suggest that these two markers are related to the same cells or bear the same relationship with the total tumour load.

The value of Cyfra 21.1 for disease monitoring could be evaluated in 23 patients. When the cases with lead time were included a concordance between clinical evaluation according to WHO response criteria and evaluation according to changes in the marker levels of 74% was observed. Most of the discordant evaluations were caused by patients who achieved minor regression or a partial response to chemotherapy while the marker level dropped below the cut-off level. When the cases in which the clinical evaluation was partial response while a normalisation of the marker took place were alone not considered to be discordant, the percentage of discordant evaluations drops to 9%. Progression of the marker with clinical stable disesae was observed only once. The possible explanation for this occurrence was a positive lead time of the marker in a patient with stable disease who was not evaluable for further response evaluation because of subsequent radiotherapy. In all the other cases a 40% increase in the level of Cyfra 21.1 indicated disease progression.

In conclusion, Cyfra 21.1 seems to be a valuable tumour marker for disease monitoring at least in patients with squamous cell lung cancer, especially since increasing levels of this marker usually indicated disease progression, and such knowledge obtained in an easy way may prevent continuation of ineffective toxic treatment.

### References

AMERICAN JOINT COMMITTEE ON CANCER (1988). In *Manual for Staging of Cancer*, Beahrs, O.H., Henson, D.E., Hutter, R.V. & Myers, M.H. (eds) pp. 115–122. Lippincott: Philadelphia.

BAC, D.J., SPLINTER, T.A.W., KOK, T.C. & VAN DER GAAST, A. (1991). Evaluation of CA19-9 serum levels for monitoring disease activity during chemotherapy of pancreatic adenocarcinoma. *J. Cancer Res. Clin. Oncol.*, **117**, 263–265.

BARTEK, J., TAYLOR-PAPADIMITRIOU, J., MILLER, N. & MILLIS, R. (1985). Patterns of expression of keratin 19 as detected with monoclonal antibodies in human breast tissues and tumors. *Int. J. Cancer*, **36**, 299–306.

BATES, S.E. (1991). Clinical applications of serum tumor markers. *Ann. Intern. Med.*, **115**, 623–638.

GEIGER, B. (1987). Intermediate filament: looking for a function. *Nature*, **329**, 392–393.

MINNA, J.D., PASS, H., GLATSTEIN, E. & IHDE, D.C. (1989). Cancer of the lung. In *Cancer: Principles & Practice of Oncology*, DeVita, W.T., Hellman, S. & Rosenberg, S.A. (eds) pp. 591–705. Lippincott: Philadelphia.

MOLL, R., FRANKE, W.W., SCHILLER, D.L., GEIGER, B. & KREPLER, R. (1992). The catalog of human cytokeratins: patters of expression in normal epithelia, tumors and cultured cells. *Cell*, **31**, 11–24.

PUJOL, J.L., GRENIER, J., DAURES, J.P., DAVER, A., PUJOL, H. & MICHEL, F.B. (1993). Serum fragment of cytokeratin subunit 19 measured by CYFRA 21-1 immunoradiometric assay as a marker of lung cancer. *Cancer Res.*, **53**, 61–66.

RASTEL, D., RAMALOLL, A., CORNILLIE, F. ON BEHALF OF THE CYFRA 21-1 MULTICENTER STUDY GROUP. (1993). CYFRA 21-1, a sensitive and specific new tumour marker for squamous cell lung cancer. Report of the First European multicenter evaluation. *Eur. J. Cancer* (in press).

SOUQUET, P.J., CHAUVIN, F., BOISSEL, J.P., CELLERINO, R., CORMIER, Y., GANZ PA, KAASA, S., PATER, J.L., QUOIX, E., RAPP, E., TUMARELLO, D., WILLIAMS, J., WOODS, B.L. & BERNARD, J.P. (1993). Polychemotherapy in advanced non small cell lung cancer: a meta-analysis. *Lancet*, **342**, 19–21.

STASIAK, P.C., PURKIS, P.E., LEIGH, I.M. & LANE, E.B. (1989). Keratin 19: predicted amino acid sequence and broad tissue distribution suggests it evolved from keratinicyte keratins. *J. Invest. Dermatol.*, **92**, 707–716.

WHO (1979). *Handbook of Reporting Results of Cancer Treatment*. Offset publication No. 48. World Health Organization: Geneva.